# Phloroglucinol and Terpenoid Derivatives from *Hypericum cistifolium* and *H. galioides* (Hypericaceae)

**DOI:** 10.3389/fpls.2016.00961

**Published:** 2016-07-04

**Authors:** Sara L. Crockett, Olaf Kunert, Eva-Maria Pferschy-Wenzig, Melissa Jacob, Wolfgang Schuehly, Rudolf Bauer

**Affiliations:** ^1^Department of Pharmacognosy, Institute of Pharmaceutical Sciences, University of GrazGraz, Austria; ^2^Department of Pharmaceutical Chemistry, Institute of Pharmaceutical Sciences, University of GrazGraz, Austria; ^3^National Center for Natural Products Research, Research Institute of Pharmaceutical Sciences, School of Pharmacy, University of Mississippi, UniversityMS, USA

**Keywords:** *Hypericum*, Hypericaceae, section *Myriandra*, anti-inflammatory, anti-bacterial, phloroglucinol, terpenoid

## Abstract

A new simple phloroglucinol derivative characterized as 1-(6-hydroxy-2,4-dimethoxyphenyl)-2-methyl-1-propanone (**1**) was isolated from *Hypericum cistifolium* (Hypericaceae) as a major constituent of the non-polar plant extract. Minor amounts of this new compound, in addition to two known structurally related phloroglucinol derivatives (**2** and **3**), and two new terpenoid derivatives characterized, respectively, as 2-benzoyl-3,3-dimethyl-4*R*,6*S*-bis-(3-methylbut-2-enyl)-cyclohexanone **(4a)** and 2-benzoyl-3,3-dimethyl-4*S*,6*R*-bis-(3-methylbut-2-enyl)-cyclohexanone **(4b)**, were isolated from a related species, *H. galioides* Lam. The chemical structures were established using 2D-NMR spectroscopy and mass spectrometry. These compounds were evaluated *in vitro* for antimicrobial activity against a panel of pathogenic microorganisms and anti-inflammatory activity through inhibition of COX-1, COX-2, and 5-LOX catalyzed LTB_4_ formation.

## Introduction

The genus *Hypericum* L. (St. John’s wort, Hypericaceae), one of the 100 largest flowering plant genera worldwide, contains to date 490 species that have been divided into 36 taxonomic sections (Hypericum Online, 2016). More than 50 native species of *Hypericum* occur in North America, of which 29 belong to the taxonomic section *Myriandra* (Sprach) R. Keller (Robson, 2003). These species, which are distributed from eastern Canada southward to Honduras and Barbados and westward to Iowa, all possess clear- to amber-colored punctate glands concentrated on the stem, leaf, sepal and petal margins. Clusters of cells that contain waxy hydrocarbons and the naphthodianthrone pigments (i.e., pseudohypericin and hypericin) and appear as minute, reddish- to black-colored glands are present in many other *Hypericum* species, including the medicinally important species *H. perforatum* L. (common St. John’s wort), but are lacking in species of section *Myriandra* (Robson, 1996). A carefully detailed anatomical study of these dark “glands” in *H. perforatum* has been conducted by Ciccarelli et al. (2001). The translucent glands, meanwhile, have been identified as the accumulation sites of acylphloroglucinol derivatives (i.e., hyperforin), compounds of significant interest due to their antidepressant, antibacterial, and anti-inflammatory activities (Beerhues, 2006; Soelberg et al., 2007).

*Hypericum cistifolium* Lam. is a shrubby or sub-shrubby representative of *Hypericum* section *Myriandra*, which branches only in its inflorescence and possesses numerous, bright-yellow flowers that are up to 12 mm in diameter. The plant is found in moist soil in pine flatwoods; along bog, swamp and marsh margins; in roadside ditches; along road embankments and generally occurs in sandy soils throughout the coastal plain of the United States from North Carolina to Louisiana. On the basis of morphological evidence, it is considered to be derived from *H. prolificum* L., a species found on calcareous and granitic soils in the eastern US. *H. galioides* Lam. is a more highly branching shrub with a rounded aspect, which is distributed in wet habitats such as stream banks, river and lake margins, swamps, flood plains, roadside ditches and low-lying pine forests throughout the coastal plain south of North Carolina and extending west to eastern Texas. Morphologically, *H. galioides* is most similar to *H. densiflorum* Pursh, a wetland plant that occurs throughout the Appalachian mountain range (Robson, 1996).

Molecular studies using sequences of the internal transcribed spacer (ITS) region of nuclear ribosomal DNA have indicated that, within *Hypericum*, species of section *Myriandra* are most closely related to those of section *Brathys sensu lato* (Mutis ex L.f.) Choisy. Species in these sections generally display shrubby or herbaceous habits, with only a few annual members represented, and possess the shared morphological characteristics of a stellate corolla, yellow petals (persistent in *H*. sect *Brathys*, but deciduous in *H*. sect. *Myriandra*), strictly pale glands, parietal placentation (incompletely axile in some *H*. sect. *Myriandra* species) and stamens in a ring (narrow or with modifications in *H*. sect *Brathys*, but broad in *H*. sect. *Myriandra*). Members of both sections lack staminodes (Nürk et al., 2012). In two separate studies, both a parsimony analysis (using *Clusia rosea* L. as an outgroup taxon) and a Bayesian analysis (including outgroup taxa from *Vismia* Vand., *Harungana* Lam. and *Cratoxylum* Blume) of ITS sequence data grouped *H. cistifolium* in a clade with *H. hypericoides* (L.) Cr., *H. tetrapetalum* Lam., *H. microsepalum* (Torrey & Gray) A. Gray ex S. Watson, *H. nudiflorum* Michx. ex Willd. and *H. apocynifolium* Small (Crockett et al., 2004; Nürk et al., 2012). Nürk et al. (2012) assigned *H. galioides* to a broader clade that contained numerous other species of section *Myriandra*, with lower bootstrap support, but indicated a sister group relationship between this species and *H. adpressum* W.P.C.Barton. Evidence for this same sister relationship had also previously been demonstrated by Crockett et al. (2004).

Relatively few chemical investigations for the purpose of elucidating taxonomic relationships within *Hypericum* have been published, and the phytochemistry of species belonging to section *Myriandra* is, in general, poorly known. The caffeoylquinic acids neochlorogenic acid, chlorogenic acid and 4-*O*-caffeoylquinic acid and the flavonoids hyperoside and isoquercitrin have been detected using HPLC in samples of fresh vegetative material of *H. cistifolium* collected in Liberty County, FL, USA, while neochlorogenic acid, rutin, isoquercitrin, quercitrin, and quercetin were detected in fresh floral material (Crockett, unpub. results). Interestingly, the caffeoylquinic acids, but not the flavonoids, were detected in an extract from dried material collected in Tallahassee County, FL, USA (Crockett et al., 2005, and Crockett, unpub. results). Compounds have not been previously isolated from this species.

When fresh vegetative material of *H. galioides* was analyzed using HPLC for these compounds, all three caffeoylquinic acids, rutin, isoquercitrin, and quercitrin were detected in samples collected from one population growing in Bryan County, GA, USA, but the caffeoylquinic acids were absent from a neighboring population growing a few miles away in the same county. Neochlorogenic acid, 4-*O*-caffeoylquinic acid and the flavonoids found in the vegetative material, as well as hyperoside, were detected in fresh floral material collected from both these populations (Crockett, unpub. results). These results highlight the difficulties inherent in the use of caffeoylquinic acids as biomarkers in *Hypericum*. Quercimeritrin, hyperoside, isoquercitrin, and quercitrin have been detected in extracts of dried material of *H. galioides* (Shakirova et al., 1972; Crockett et al., 2005). In an examination of the volatile constituents of the aerial material, caryophyllene oxide (12.9%) and guaia-6,10(14)-dien-4*β*-ol (18.5%) were identified as major components of the distilled volatiles (Crockett et al., 2008a). Again, however, compounds have not been previously isolated from this species. During the study by Crockett et al. (2005), several late-eluting peaks were observed that had UV spectral pattern characteristics compatible with those of phloroglucinol derivatives, prompting the current investigation (pers. obs.). The high level of structural diversity inherent among phloroglucinol derivatives, coupled with their bioactivities, makes this class of substances an interesting target of phytochemical research.

Considering the known bioactivities of reported phloroglucinol derivatives, the anti-inflammatory potentials of extracts and isolated compounds from *H. cistifolium* and *H. galiodes* were determined using *in vitro* assays that measured the inhibition of COX and 5-LOX product formation. COX-1, COX-2, and 5-LOX are the key enzymes of arachidonic acid metabolism that lead to the production of important mediators of inflammation. COX-1 and -2 catalyze the first two steps in prostaglandin synthesis and 5-LOX catalyzes the oxygenation of arachidonic acid in the first step of the leukotriene pathway. In addition, antimicrobial screening using an *in vitro* microplate assay was conducted to target extracts and fractions with antibacterial and/or antifungal activity. This work resulted in the characterization of one new phloroglucinol and two new terpenoid derivatives.

## Materials and Methods

### General Experimental Procedures

Polarimetry was performed on a Perkin-Elmer 241-MC polarimeter, in a 10-cm microcuvette. FTIR data were acquired using a Perkin-Elmer spectrometer, model-spectrum BX-series, in cm^-1^ (PerkinElmer, USA). CD measurements in MeOH were carried out on a Jasco J-715 spectropolarimeter (Welltech Enterprises, Inc., USA) using a 0.1 cm path-length cell (λ_range_ 200–400 nm, resolution 0.2 nm, scan speed 50 nm/min, T 25°C, five scans averaged). ^1^H-, ^13^C-, and 2D-NMR experiments (HSQC, HMBC, DQF-COSY) were performed with Varian Unity Inova -400 and -600 MHz spectrometers. Chemical shift values (δ) were reported in ppm relative to tetramethylsilane (TMS, δ = 0) as an internal standard and coupling constants (*J-*values) are given in Hertz (Hz). The compounds **1–4** were dissolved in CDCl_3_ and spectra were recorded at 25°C. Experimental parameters were as published in Seebacher et al. (2003). HPLC-DAD/ESI-MS (neg.) data were obtained on a Thermo Finnigan Surveyor LC instrument with Thermo Quest Surveyor DAD, autosampler, and MS pump, and a Thermo Finnigan LCQ-XP mass detector equipped with an ESI source run by Xcalibur software (Thermo Finnigan, USA). Analytical HPLC was performed using a Zorbax SB RP-18 column (3 μm, 2.1 × 150 mm; Agilent Technologies), flow rate 250 μL/min, gradient elution H2O/MeOH (25:75 to 0:100 over 20 min, 10 min at 0:100, 10 min equilibration). Mass spectra were detected and recorded (scan range = *m/z* 50–1000, transfer capillary temperature = 350°C, spray voltage = 5.00 kV, sheath gas flow = 70 units). VLC was performed on either fine-grade silica gel 60 (230–400 mesh ASTM; Merck) or medium-grade silica gel 60 (70–230 mesh ASTM; Merck). Analytical TLC was performed on silica gel 60 F_254_ plates (Merck), eluting with hexane/EtOAc 75:25, visualization by spraying with H_2_SO_4_ (10% solution v/v in 95% aq. EtOH) and then vanillin (5% solution w/v in 95% aq. EtOH) reagents, then heating at 150° for 45 s and detecting under UV/VIS light at 254 and 365 nm. Preparative HPLC and detection of UV spectra (λ_max_ in nm) were performed on an Agilent 1100 Separations Module equipped with a photodiode array detector (Agilent Technologies, USA), using a LiChroCART RP-18 column (LiChrospher, 7 μm, 10 × 250 mm; Merck), flow rate 2 mL/min, MeCN:H_2_O gradient system (50:50 to 100:0 over 30 min), detection at 254 and 280 nm.

### Plant Material

Aerial material of *H. cistifolium* Lam. (Hypericaceae) was collected from Tallahassee County, FL, USA. A voucher specimen (Crockett-H65) has been deposited at the University of Georgia (UGA) Herbarium in Athens, GA. Aerial material of *H. galioides* Lam. was collected from Liberty County, FL, USA. A voucher specimen (Crockett-152B) has been deposited at the University of Mississippi (UMISS) Herbarium in Oxford, MS, USA. The aerial material (flowers, inflorescence bracts, and upper stems) was collected while plants were in full flower, and plants were identified by S. Crockett. Material was dried in darkened, ventilated cabinets to a moisture content of less than 2% prior to grinding.

### Extraction and Isolation

*Hypericum cistifolium*: 800 g aerial material was ground and extracted in a maceration tank with CH_2_Cl_2_ (4 L, 5 days). 12.5 g extract was further investigated due to interesting TLC features (i.e., a bright orange band, following spraying and heating). 10 g of the CH_2_Cl_2_ extract was subjected to VLC using fine-grade silica (100 g), eluting with a hexane-EtOAc gradient (5% steps, 200 mL/step, 25 mL fractions). A bright yellow band eluted from the column with the first 400 mL solvent (<5% EtOAc in hexane). Upon sitting overnight, a clear crystalline material precipitated from these initial fractions. This substance was purified by repeated crystallization from hexane, yielding 985 mg (∼10.2% w/w yield) of compound **1**. *H. galioides*: 940 g aerial material was ground and extracted in a maceration tank with *n*-hexane (5 L, 5 days), yielding 13.5 g extract. TLC examination of this extract revealed several bright orange- and blue-staining bands upon spraying with a vanillin/sulphuric acid reagent and heating. 13 g of this extract was subjected to VLC on fine-grade silica (150 g), eluting with a hexane-EtOAc gradient (5% steps, 300 mL/step, 150 mL fractions) to yield six fractions (after combining on the basis of TLC similarities). A bright yellow visible band was observed eluting from the column with 20% EtOAc, and this fraction (ca. 1 g) was further purified by VLC on medium-grade silica (100 g), using a hexane-Et_2_O gradient (10% steps, 200 mL/step, 25 mL fractions), yielding 10 fractions. The MeCN-soluble portion of merged fractions 3–5 were further purified by preparative RP-HPLC, resulting in the isolation of one new (**1**) and two known (**2** and **3**) phloroglucinol derivatives, in amounts of 3.5, 12, and 2 mg, respectively. In addition, 4 mg of a known compound (4,12,12-trimethyl-9-methylene-5-oxatricyclo[8.2.0.04,6] dodecane or *β*-caryophyllene oxide) was isolated. This compound was identified by comparison of its 1H- and 13C-NMR and mass values with those cited in literature (Harper et al., 1998).

On the basis of interesting TLC bands (yellow-green fluorescence under UV_366_, turning blue when sprayed and heated), the fraction eluting from the original VLC column with 30% EtOAc was additionally selected for further purification. This extract (ca. 1 g) was subjected to VLC on medium-grade silica (100 g), eluting with a hexane-EtOAc gradient (5% steps, 200 mL/step, 25 mL fractions), to yield 10 fractions. Fractions 3–5 were merged on the basis of TLC similarities and repeated crystallization from hexane:EtOAc (75:25) yielded compound **4** (16 mg, 0.02% w/w), which was subsequently characterized as a racemic mixture of two new terpenoid derivatives (**4a** and **4b**).

**1-(6-Hydroxy-2,4-dimethoxy-phenyl)-2-methyl-1-propanone** (**1**) Clear crystalline solid; UV (CH_2_Cl_2_) λ_max_ (log ε): 287 (5.47) nm; IR (thin film) ν_max_ 3447, 2977, 1626, 1222 cm^-1^; ^1^H-NMR and ^13^C-NMR (MeOH-d4): see **Table 1**; ESI-MS (*m/z*): 224.1 (calc. for C_11_H_14_O_4_, 224.1049).

**Table 1:** ^1^H and ^13^C NMR chemical shifts (ppm) of compounds **1** – **3** in MeOH-d_4_ at 25°C, TMS as internal standard, *J* in Hz.

	1			2			3		
			
Atom	_δC_		_δH_	_δC_		_δH_	_δC_		_δH_
1	106.4		-	106.9		-	110.3		-
2	167.7		-	168.1		-	161.1		-
3	91.9		6.07 *s*	92.1		6.06 *s*	112.7		-
4	164.1		-	164.6		-	165.4		-
5	95.3		6.06 *s*	95.3		6.06 *s*	96.8		6.28 *s*
6	168.1		-	168.1		-	163.9		-
									
2-*O*Me	56.1		3.82 *s*	56.2		3.82 *s*	63.0		3.68 *s*
3-Me							19.0		2.04 *s*
4-*O*Me	56.3		3.88 *s*	56.4		3.88 *s*	56.1		3.83 *s*
									
1′	211.7		-	207.4		-	212.3		-
2′	40.8		3.76 *sept.*, *J* = 6.6	47.4		2.96 *t*, *J* = 7.2	40.5		3.76 *sept.*, *J* = 6.6
3′	19.6		1.12 *d*, *J* = 6.6	19.7		1.67 *sext.*, *J* = 7.2	20.0		1.12 *d*, *J* = 6.6
4′	19.6		1.12 *d*, *J* = 6.6	14.6		0.98 *t*, *J* = 7.2	20.0		1.12 *d*, *J* = 6.6


**2-Benzoyl-3,3-dimethyl-4*R*,6*S*-bis-(3-methylbut-2-enyl)-cyclohexanone** (**4a**) and **2-Benzoyl-3,3-dimethyl-4*S*,6*R*-bis-(3- methylbut-2-enyl)-cyclohexanone** (**4b**) Pale yellow solid; UV (CH_2_Cl_2_) λ_max_ (log ε): 243 (3.55) nm; IR (thin film) ν_max_ 3395, 2922, 1718, 1655, 1463 cm^-1^; ^1^H-NMR and ^13^C-NMR (CDCl3): see **Table 2**; ESI-MS (*m/z*): 398.3 (calc. for C_27_H_42_O_2_, 398.3185).

**Table 2:** ^1^H and ^13^C NMR chemical shifts (ppm) of compound **4** in CDCl_3_ at 25°C, TMS as internal standard, *J* in Hz.

Atom	_δC_	_δH_	atom	_δC_	_δH_
1	207.8	-	1′	196.7	-
2	67.3	4.39 *s*	2′	138.8	-
3	44.6	-	3′	127.7	7.78 *d*, *J* = 7.8
4	48.8	1.70	4′	128.5	7.42 *t*, *J* = 7.8
5	35.4	1.25, 2.20 *ddd*, *J* = 14.2, 6.4, 3.5	5′	132.7	7.52 *t*, *J* = 7.6
6	51.4	2.51 *sext*., *J* = 6.4	6′	128.5	7.42 *t*, *J* = 7.8
7	26.8	1.13 *s*	7′	127.7	7.78 *d*, *J* = 7.8
8	16.1	1.15 *s*			
1″	27.9	1.72 *m*, 2.26 *m*	1^′′′^	27.7	1.97 *dtr*, *J* = 14.8, 6.5, 2.41 *dtr*, *J* = 14.6, 6.0
2″	123.2	5.17 *t*, *J* = 6.8	2^′′′^	121.6	5.09 *t*, *J* = 6.8
3″	132.8	-	3^′′′^	133.2	-
4″	17.9	1.62 *s*	4^′′′^	17.9	1.60 *s*
5″	25.8	1.74 *s*	5^′′′^	25.8	1.68 *s*


## Bioassay Testing

*In vitro* assays for COX-1 and COX-2 enzymatic inhibitory activity were performed in a 96-well-plate format with purified prostaglandin H synthase (PGHS)-1 from ram seminal vesicles for COX-1 and purified PGHS-2 from sheep placental cotyledons for COX-2 (both Cayman Chemical Company, Ann Arbor, MI, USA). The bioassay for inhibition of leukotriene formation was carried out in 96-well-plate format using polymorphic leukocytes with 5-LOX activity, isolated from venous human blood. Further details of the bioassays for anti-inflammatory activity are described in Crockett et al. (2008b). Antimicrobial testing using an *in vitro* microplate assay were performed as reported in Samoylenko et al. (2009).

## Results and Discussion

**FIGURE 1:**
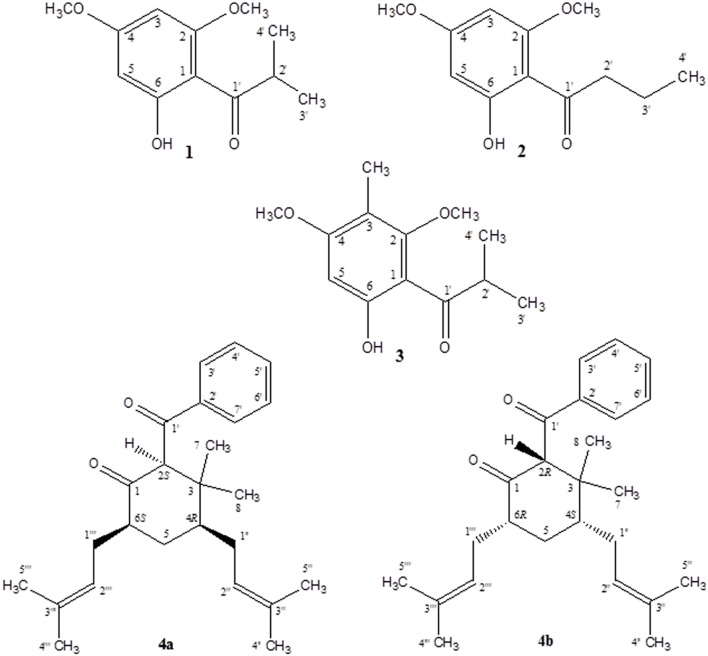
**Compounds isolated from *H. cistifolium* and *H. galioides***.

As part of our continuing phytochemical investigation of the genus *Hypericum*, and in particular, the identification and elucidation of chemotaxonomic markers in species of section *Myriandra*, the non-polar extracts of *H. cistifolium* and *H. galioides* were targeted on basis of interesting TLC, UV, and HPLC characteristics. Previously, we had observed that simple phloroglucinols and filicinic acid derivatives generally display red to orange colors during TLC analysis upon spraying and heating, while more complex structures (e.g., those with bicyclo[3.3.1]nonane base structures) display blue to purple colors (Crockett, pers. obs.). These characteristics aided the chromatographic fractionation and purification of phloroglucinol and terpenoid derivatives, namely, three new (**1**, **4a,** and **4b**) and two known compounds (**2** and **3**), which were subsequently identified from these two *Hypericum* species (**Figure 1**).

Despite the structurally simple nature of compound **1**, it has not been previously reported in the scientific literature to our knowledge. This compound occurred as a major component of the non-polar extract of *H. cistifolium* (>10% w/w) and was isolated from *H. galioides* as a minor constituent (0.03% w/w) as a clear, crystalline solid with a pleasant odor. Its structure was established through NMR spectroscopy. The ^1^H- and ^13^C-NMR spectra indicated an asymmetrically tetrasubstituted phenyl group with two methoxy, one hydroxyl and one isobutyryl moiety, with methoxy and hydroxyl groups on alternating carbons. The relative positions of the substituents were determined using HMBC correlations. Both aromatic protons coupled to C-1 (106 ppm), but only the one at 6.07 ppm coupled to both carbons attached to the methoxy groups.

Compound **2** (0.09% w/w) has been both synthesized (Canter et al., 1931) and isolated from a natural plant source as a pale, crystalline substance with no remarkable scent from two species of *Dysophylla* (Lamiaceae) (Joshi and Ravindranath, 1977; Nanda et al., 1983). Interestingly, this compound is used as a starting reagent in the synthesis of (+)-calanolide-A, a coumarin that was originally isolated from another member of Calophyllaceae (Clusiaceae *sensu lato*), *Calophyllum lanigerum* var. *austrocoriaceum*, and that has been investigated as a potential anti-HIV drug candidate in the USA (Tanaka et al., 2000). Compound **3** (0.02% w/w) was identified as a known substance that has been previously synthesized (Schiemenz et al., 1985) and isolated from the leaf oils of several species in the plant family Myrtaceae including *Thryptomene saxicola* (Dastlik et al., 1989), *Austromyrtus dulcis* (Brophy et al., 1995), *Eucalyptus miniata* (Ireland et al., 2004), and *Xanthostemon eucalyptoides* (Brophy et al., 2006). This substance has been given the informal name isobaeckeol and has been described in previous literature as a light pink substance with a faint pleasant odor. The identities of compounds **2** and **3** were verified by comparison with values reported in the literature (**2**: Joshi and Ravindranath, 1977; Äyras and Widén, 1978 and **3**: Schiemenz et al., 1985; Ireland et al., 2004). Because complete data sets for these compounds have not been previously published, we include a comparison of the values for compounds **1**–**3** here in **Table 1**.

A simple acylphloroglucinol with a very similar structure to compounds **1**–**3** has also been isolated from *H. beanii* N. Robson (section *Ascyreia*, Shiu and Gibbons, 2006). While this compound demonstrated moderate activity (MIC = 16–32 μg/mL) against a panel of multidrug-resistant strains of *Staphylococcus aureus*, compound **1** tested in the same panel (data not shown) displayed no anti-staphylococcal activity. The compound isolated from *H. beanii* differs from compound **1** only in that it possesses a hydroxyl group at C-5 (rather than a methoxy group) and a methylation at C-6 (see **Figure 1**), indicating that modification at these positions have the potential to influence anti-staphylococcal activity. Interestingly, prenylated phloroglucinol derivatives that contain sub-structures of compounds **1**–**3** have been previously isolated from a Jamaican collection of *H. hypericoides*, which has been hypothesized to be closely related to *H. cistifolium* (Christian et al., 2008; Nürk et al., 2012). An analysis of other species in the section Myriandra revealed the presence of peaks eluting in or near the same region as compound **1** (e.g., *H. brachyphyllum, H. densiflorum, H. edisonianum, H. hypericoides, H. lissophloeus, H. lobocarpum, H. prolificum, H. suffruticosum*), and further chemical investigations of these species have the potential to yield additional phloroglucinol derivatives (Crockett et al., 2005; Henry et al., 2006).

Compounds **4a** and **4b** have not been previously reported as natural products isolated from *Hypericum* or another plant species to our knowledge. These compounds were isolated as a mixture, a pale-yellow odorless solid, representing minor constituents (0.02% w/w) of the lipophilic extract of *H. galioides*. ^1^H- and ^13^H-NMR spectral analyses revealed that compound **4** consisted of a six-membered ring with a benzophenone group at C-2, two methyl groups at C-3, and isoprenyl groups at C-4 and C-6. C-1 was represented by a keto group and C-5, by a methylene group. The relative configurations at the stereogenic centers were determined through analysis of cross-peaks in COSY and enhancements in selective 1D NOE spectra. Selective inversion of H-2 (4.39 ppm) led to NOEs at H-4 and H-6, indicating an axial orientation for all these protons. An additional NOE between H-2 and a signal at 1.15 ppm allowed the assignments of the methyl groups bound to C-3. Evidence for the equatorial orientation of H-5 (1.25 ppm) is supported by the coupling constants (*J* = 3.5, 6.4, and 14.2 Hz), although the multiplicity of the signal was obscured by methylene resonances from a minor fatty acid constituent in the sample. The axial orientations of H-4 and H-6 were confirmed by strong COSY cross-peaks to the equatorial proton H-5. The ‘sexet’ reported for H-6 arises from the superposition of a doublet (*J* ≈ 13 Hz) of quadruplets (*J* ≈ 6.5 Hz) with relative intensities of 1:3:4:4:3:1. A comparison with NOE-derived data (not shown) was performed to assign the resonances of the methyl groups in the isoprene side-chains.

**FIGURE 2:**
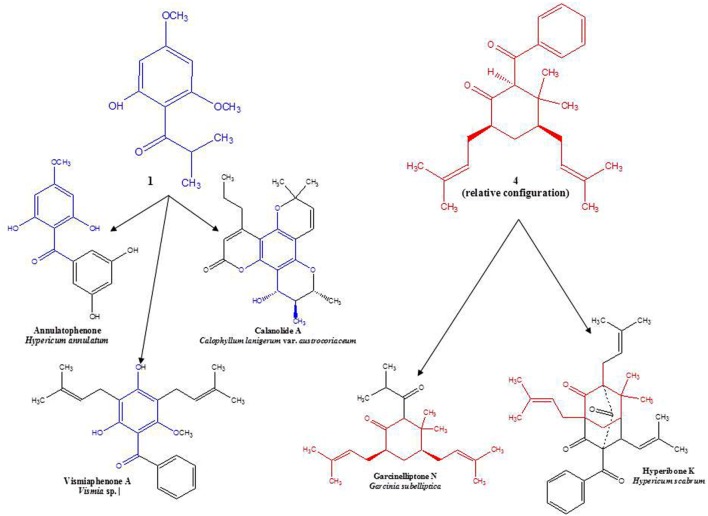
**Examples of structural similarities between currently and previously isolated compounds from selected species of Hypericaceae, Calophyllaceae, and Clusiaceae (Delle Monache et al., 1980; Kitanov and Nedialkov, 2001; Tanaka et al., 2004; Weng et al., 2004)**.

Circular dichroism spectra were taken to determine the absolute configuration at C-2, -4, and -6 in the molecule. However, no CD signal was detected, indicating the presence of a racemic mixture of **4a** and **4b**. Interestingly, this structure occurs wholly or in part as a sub-structure within other phloroglucinol derivatives that have been isolated from certain members of the family, Clusiaceae, which is related to Hypericaceae. A similar observation may be made for the base structure of compounds **1**–**3** (see **Figure 2**). Along with data from literature, these findings support hypotheses for biogenetic links among several sections of *Hypericum*, as well as between *Hypericum* and other currently recognized tribes in Hypericaceae (i.e., Vismieae and Cratoxyleae), and between Hypericaceae and other related families, namely Clusiaceae *nom. cons.* and Calophyllaceae, within the broader plant order Malpighiales.

As several phloroglucinol derivatives isolated from *Hypericum* species are known to possess anti-inflammatory and antibacterial activities, the crude CH_2_Cl_2_ extracts as well as the isolated new compounds were subjected to antibacterial and anti-inflammatory *in vitro* testing: CH_2_Cl_2_ extracts of *H. cistifolium* and *H. galioides* were submitted to the National Center for Natural Products Research (NCNPR, Oxford, MS, USA) for bioassay testing against a variety of pathogenic microorganisms including the fungi *Candida albicans*, *C. glabrata*, *C. krusei*, *Cryptococcus neoformans*, *Aspergillus fumigatus*, and the bacteria *Mycobacterium intracellulare*, *Pseudomonas aeruginosa*, *S. aureus*, and methicillin-resistant *S. aureus* (MRSa). Only the extract of *H. cistifolium* displayed a marginal activity against *S. aureus* (MRSa) and *C. neoformans* with IC_50_s of 100–150 μg/mL. In addition, when tested at 50 μM *in vitro* against enzymes involved in arachidonic acid metabolism, compounds **1** and **4a/b** displayed negligible or only very weak bioactivity against COX-1, COX-2, and 5-LOX product formation.

The new compounds isolated from two species of *Hypericum* section *Myriandra* displayed relatively low bioactivity levels in the bioassays selected, although structurally similar compounds isolated from other species have demonstrated higher levels of activity, and the evidence collected thus far seems to indicate that the degree of hydroxylation and methylation, as well as the positions of these moieties, can strongly influence bioactivity. Commercially, *H. cistifolium* and *H. galioides* occupy a very small niche market within the horticultural industry and are sold as ornamental landscaping plants, primarily in the southeastern United States (Huxley et al., 1992). Because established cultivation methods for these species exist, however, larger-scale production for the purposes of phytochemical isolation of these compounds could be rapidly established.

While phloroglucinol derivatives have been previously isolated as both major and minor constituents from many species of *Hypericum*, as well as related genera within Clusiaceae *sensu lato*, their ecological roles in the plants are still poorly understood. Hypotheses suggesting that such compounds act as attractive (for pollinators) and/or defensive (against herbivores) substances in the plant have been proposed, but further studies are needed (Gronquist et al., 2001). The potential ecological role of such compounds as compound **1**, produced in such high amounts by *H. cistifolium*, is an area of considerable research interest for our group. Phylogenetic hypotheses have been proposed regarding relationships between and among species in section *Myriandra* on the basis of morphological and molecular evidence (Robson, 1996; Crockett et al., 2004; Nürk et al., 2012). Steadily accumulating evidence from phytochemical investigations indicates that acylated and prenylated phloroglucinol derivatives are not only compounds of significant interest due to their respective bioactivities, but also have the potential to be used as chemotaxonomic markers in *Hypericum*. Future directions of research include the phytochemical investigation of species (i.e., *H. hypericoides*, *H. tetrapetalum*, *H. microsepalum*, *H. nudiflorum*, *H. apocynifolium*, and *H. adpressum*) closely allied to those in the current investigation.

## Author Contributions

SC, plant collection, compound isolation (TLC and CC), HPLC-DAD/ESI-MS analysis, manuscript preparation and revision; OK, NMR; EP and RB, *in vitro* assays for assessing COX-1 and COX-2 enzymatic inhibitory activity and inhibition of leukotriene formation (5-LOX activity); MJ, *in vitro* antimicrobial testing; WS, circular dichroism measurements.

## Conflict of Interest Statement

The authors declare that the research was conducted in the absence of any commercial or financial relationships that could be construed as a potential conflict of interest.

## Abbreviations

CD: circular dichroism
COSY: correlation spectroscopy
COX: cyclooxygenase
DAD: diode array
DFQ: double quantum-filled
ESI: electrospray ionization
FTIR: fourier transform infrared
HMBC: heteronuclear multiple bond correlation
LOX: lipoxygenase
LTB_4_: leukotriene B_4_
NOE: nuclear overhauser effect
